# Effect of fluvalinate on the expression profile of circular RNA in brain tissue of *Apis mellifera ligustica* workers

**DOI:** 10.3389/fgene.2023.1185952

**Published:** 2023-05-11

**Authors:** Shan Xueqing, Lou Delong, Wang Guizhi, Fan Yunhan, Yang Liuxu, Chao Tianle

**Affiliations:** ^1^ Shandong Provincial Key Laboratory of Animal Biotechnology and Disease Control and Prevention, Shandong Agricultural University, Tai’an, Shandong, China; ^2^ Comprehensive Testing and Inspection Center, Shandong Provincial Animal Husbandry and Veterinary Bureau, Jinan, Shandong, China; ^3^ Key Laboratory of Efficient Utilization of Non-grain Feed Resources (Co-construction by Ministry and Province), Ministry of Agriculture and Rural Affairs, Shandong Agricultural University, Tai’an, Shandong, China

**Keywords:** *Apis mellifera ligustica*, circular RNAs, fluvalinate, brain tissue, ceRNA

## Abstract

Fluvalinate is widely used in apiculture as an acaricide for removing Varroa mites, but there have been growing concerns about the negative effects of fluvalinate on honeybees in recent years. Previous research revealed changes in the miRNA and mRNA expression profiles of *Apis mellifera ligustica* brain tissues during fluvalinate exposure, as well as key genes and pathways. The role of circRNAs in this process, however, is unknown. The goal of this study was to discover the fluvalinate-induced changes in circular RNA (circRNA) expression profiles of brain tissue of *A. mellifera ligustica* workers. A total of 10,780 circRNAs were detected in *A. mellifera ligustica* brain tissue, of which eight were differentially expressed between at least two of the four time periods before and after fluvalinate administration, and six circRNAs were experimentally verified to be structurally correct, and their expression patterns were consistent with transcriptome sequencing results. Furthermore, ceRNA analysis revealed that five differentially expressed circRNAs (DECs) (novel_circ_012139, novel_circ_011690, novel_circ_002628, novel_circ_004765, and novel_circ_010008) were primarily involved in apoptosis-related functions by competitive binding with miRNAs. This study discovered changes in the circRNA expression profile of *A. mellifera ligustica* brain tissue caused by fluvalinate exposure, and it provides a useful reference for the biological function study of circRNAs in *A. mellifera ligustica*.

## 1 Introduction

*Apis mellifera ligustica* is a high-value insect species that performs plant pollination in agricultural production (Aizen and Harder, 2009; Beck et al., 2015; Andonov et al., 2019) and produces high-value byproducts such as honey and royal jelly (Berry et al., 2013). The most common bee parasite is *Varroa destructor*, which is especially harmful to *A. mellifera ligustica* (Burley et al., 2008).

Fluvalinate is a pyrethroid acaricide that is commonly used to kill *V. destructor* (Camp et al., 2010). Fluvalinate can kill *V. destructor* by affecting the nervous system’s voltage-gated sodium channels, causing overexcited nerves and paralysis (Chao et al., 2022a). Fluvalinate, on the other hand, is toxic to *A. mellifera ligustica* (Chen et al., 2020; Chao et al., 2022b). Several studies have reported negative effects of fluvalinate exposure on *A. mellifera ligustica*, with the results indicating that *A. mellifera ligustica*’s sensory and memory systems may be impaired (Choi et al., 2006; Davies et al., 2007; Du et al., 2017; Ding et al., 2022). Our previous research has revealed changes in protein-coding gene and miRNA expression profile of *A. mellifera ligustica* brains caused by fluvalinate exposure, and some differentially expressed genes and miRNAs related to apoptosis, visual function, and neural response have been identified (Fischer and Raabe, 2018; El Agrebi et al., 2019). In our two previous studies, the phototransduction pathway was the primary pathway for significant enrichment, which may indicate that the toxicological mechanism of fluvalinate is associated with visual impairment. We also predicted that key genes and miRNAs including LOC412299, LOC411188, and ame-miR-3477-5p may be involved in fluvalinate resistance (Fischer and Raabe, 2018; El Agrebi et al., 2019). However, at present, the expression profile changes and functions of circRNA during fluvalinate exposure are still unclear.

Circular RNAs are naturally occurring RNAs found in eukaryotic transcriptomes (Frost et al., 2013). CircRNAs have been shown to have a “sponge” function for miRNAs (Glažar et al., 2014; Gan et al., 2017), and this mechanism can also be used to indirectly regulate mRNA expression (Gujar et al., 2019). Several studies have demonstrated the expression of circRNAs in multiple species of honeybees (Gujar et al., 2019; Han et al., 2021), with reports on the involvement of circRNAs in the regulation of *A. mellifera ligustica* brain functions related to foraging and nursing functions (Han et al., 2021). Currently, to the best of our knowledge, there have been no reports on the function of circRNAs in the fluvalinate exposure process.

In this study, we combined transcriptome sequencing data from previous studies to detect and analyze the changes in the circRNA expression profile of *A. mellifera ligustica* brain tissue before and after fluvalinate treatment, and we built a competing endogenous RNA (ceRNA) network by integrating the differentially expressed circRNA with miRNA and mRNA for a more in-depth analysis of circRNA function.

## 2 Materials and methods

### 2.1 Ethics statement

All animal protocols used in this study were approved by the Animal Protection and Ethics Committee of Shandong Agricultural University (protocol number: SDAUA-2018-055).

### 2.2 Sample collection, total RNA extraction, and cDNA synthesis

This study followed the same overall strategy, sample collection, total RNA extraction, and cDNA synthesis as our prior investigations (Fischer and Raabe, 2018; El Agrebi et al., 2019). Six *A. mellifera ligustica* colonies were obtained from the Fuxin Apiary in Tai’an City, Shandong Province, China. All colonies had the same potential and were randomly divided into two groups (three colonies as the test group and three colonies as the control group). The test colonies were administered with a standard dose of fluvalinate. The sample honeybees were obtained 1 day before fluvalinate treatment began and 10 days, 20 days, and 30 days later, with 20 adult worker bees gathered from each colony for each sampling. Afterward, honeybee brains were dissected under a microscope, and bee brains from the same colony were pooled for total RNA extraction and cDNA synthesis. Total RNA was isolated from *A. mellifera ligustica* brain tissue samples according to the manufacturer’s instructions using TRIzol reagent (Invitrogen, Carlsbad, CA, United States). The quantity and quality of RNA were determined using an Agilent 2100 Bioanalyzer (Agilent Technologies, Palo Alto, CA, United States) and sequenced using the Illumina HiSeq TM 4000 system’s standard protocol.

### 2.3 Sequencing data filtering and alignment analysis

The transcriptome sequencing data used in this study were identical to those used in our prior work, as were the quality control data (Fischer and Raabe, 2018; El Agrebi et al., 2019). Bowtie2 (version 2.3.5.1) (Hansen et al., 2013) was used to align and exclude ribosomal reads, and TopHat2 (version 2.0.3.12) (He et al., 2017) was used to align the remaining reads to the honeybee reference genome GCF-003254395.2. Then, to obtain anchor reads, we extracted unmapped reads and intercepted both ends of unmapped reads (default 20 bp). The anchor reads were matched once again with the reference genome GCF-003254395.2, and the alignments were concatenated for circRNA identification.

### 2.4 Identification of differentially expressed circRNAs

circRNA identification was performed using find_circ (Hung et al., 2018). The identified circRNAs were aligned to the circBase (Johnson, 2015) database using BLAST, and circRNAs with the threshold of E < e−10 were identified as highly credible annotated circRNAs, otherwise new predicted circRNAs (novel_circRNAs). DESeq2 (Kanehisa and Goto, 2000) was used to detect DECs with an FDR of 0.05 and |log2(FC)| > 1.

### 2.5 Circular structure verification and qRT-PCR verification of differentially expressed circRNAs

cDNA was synthesized using the Evo M-MLV Plus cDNA synthesis kit. All the eight DECs were selected for junction region and qRT-PCR verification with their sequence-specific primers (Table 1). The qRT-PCR was performed using the SYBR^®^ Green Pro Taq HS Master Mix qPCR Kit II (Accurate Biology). qPCR reactions were performed in two steps on the Bio-Rad CFX96 Real-Time Detection System (Bio-Rad Laboratories, Inc., United States): 95°C for 30 s; 95°C for 5 s, and 60°C for 30 s, 40 cycles; and adding a dissolution curve of 95°C for 1 min, 62°C for 30 s, and 95°C for 30 s. The 2^−ΔΔCT^ method was used to calculate the relative expression levels of circRNAs. *A. mellifera ligustica* AmAct (Kim et al., 2013) was used as a control. Three biological replicates were used for each group of samples, and each biological replicate was tested with three technical replicates.

**TABLE 1:** Primer information for circRNA loop junction site verification and qRT-PCR testing.

Primer	Primer sequence (5′–3′)	Product length (nt)	Melting temperature (Tm) (°C)
novel_circ_011690-F	TAG​CGA​GCA​CAG​GAG​GTA​GCA	120	60
novel_circ_011690-R	CCG​CAA​GTG​GAG​CAT​TTG​AAA​CA		
novel_circ_002628-F	GGA​GAA​GGC​TGA​CGA​GGA​AGT	84	60
novel_circ_002628-R	TTC​ACG​TTC​AGG​CTC​GAC​AAC		
novel_circ_010008-F	GCT​TGC​TAC​ATC​CTG​ATT​ACA​CCT	176	60
novel_circ_010008-R	TTG​ACC​AGA​TTC​ATA​GAC​CCG​AAA		
novel_circ_000054-F	TCG​CTT​GCT​ACA​TCC​TGA​TTA​CAC	176	60
novel_circ_000054-R	CCA​ATA​ACG​CTG​GGA​CTA​GAA​TGA		
novel_circ_004765-F	TGC​GAT​TCA​ATC​TGG​CGA​ATA​TGA	156	60
novel_circ_004765-R	AGA​AGT​CGT​CCA​CCC​TTA​CCA		
novel_circ_012139-F	GTT​GCC​GTA​AAT​GCC​ACT​ACA	154	60
novel_circ_012139-R	GAT​GGA​AGG​AAT​CGT​CGG​AAT​T		
novel_circ_006817-F	AAG​ACC​ACC​TGG​CTC​TAG​TAC​A	131	60
novel_circ_006817-R	CTG​TGC​TAC​CTG​AAC​TGG​ATT​GT		
novel_circ_004398-F	AGT​AGC​ACA​GAA​CAA​CCA​GGT​AGT	134	60
novel_circ_004398-R	GAC​GTG​GAC​GGT​GTA​CTT​GAA​C		
AmAct_F	TGC​CAA​CAC​TGT​CCT​TTC​TG	156	60
AmAct_R	AGA​ATT​GAC​CCA​CCA​ATC​CA		

### 2.6 Co-expression analysis between mRNAs and DECs

Pearson’s correlation analysis was performed on differentially expressed circRNAs and differentially expressed mRNAs (with |r| > 0.7 and significant *p* < 0.05 as thresholds). The expression profiles of differentially expressed mRNAs were obtained from our previous research (Fischer and Raabe, 2018). A hierarchical clustering was performed with the correlation r-values using MultiExperiment Viewer (MeV) (Kozomara et al., 2019). GO and KEGG (Krüger and Rehmsmeier, 2006) enrichment analyses were performed on mRNAs associated with differentially expressed circRNAs using clusterProfiler (Langmead and Salzberg, 2012), and FDR <0.05 was used as a significant threshold.

### 2.7 Construction and analysis of the ceRNA network

The potential targeting relationships of miRNA–mRNA and miRNA–circRNA were predicted using RNAhybrid (version 2.1.2) (Lee et al., 2013), Miranda (version 3.3a), and TargetScan (version 7.0) (Lewis et al., 2005; Love et al., 2014), and the intersection of the three software results was taken as a trusted targeting relationship. We also performed Pearson’s correlation analysis between miRNAs and mRNAs with |r| > 0.7 and *p* < 0.05 as thresholds. Finally, the intersection of correlation analysis and targeting relationship results was considered potential regulatory relationships and was used for the construction of the ceRNA network using Cytoscape (version 3.8.0) (Marco and Griffiths-Jones, 2012).

## 3 Results

### 3.1 Data quality control

Similar to our previous research, 956,250,750 clean reads were obtained from the brain tissue transcriptome sequencing of *A. mellifera ligustica* after treatment with fluvalinate (Fischer and Raabe, 2018).

The high-quality clean reads were aligned to the ribosome database, and 719,788,610 ribosome unaligned reads were obtained. The ribosome unaligned reads were then mapped with the *A. mellifera ligustica* reference genome GCF-003254395.2, and 65,829,717 unmapped reads were used for anchor read construction. The anchor reads were compared to the genome GCF-003254395.2 again, and a total of 63,050,916 mapped anchor reads were obtained for further circRNA identification.

### 3.2 CircRNA identification

A total of 10,780 newly discovered circRNAs were identified, of which 10,764 circRNAs were annotated on 16 known chromosomes in the *A. mellifera ligustica* genome and 16 circRNAs were annotated to unknown chromosomal locations. It was found that 7,192 (66.72%) circRNAs were composed entirely of annotated exons, 424 (3.93%) circRNAs were composed of introns, 1,262 (11.71%) circRNAs were composed of both exons and introns, 371,287 (2.66%) circRNAs were located in intergenic regions, 1,243 (11.53%) circRNAs were composed of single exons, and only one circRNA (novel_circ_004083) belonged to chromosome type unknown.

The expression values of circRNAs were normalized as reads per million mapped reads (RPM).

### 3.3 Identification of DECs

**FIGURE 1:**
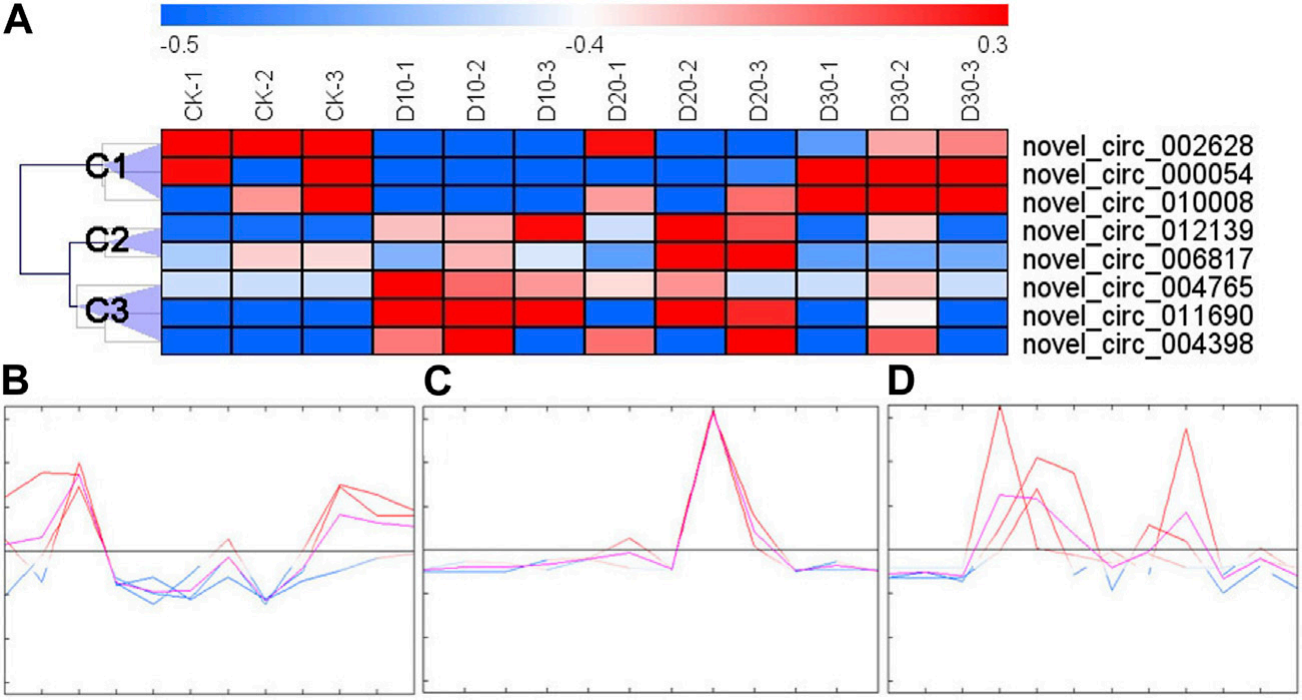
Clustering heat map and expression pattern of differentially expressed circRNAs. **(A)** The clustering heat map of the differentially expressed circRNAs shows that each column represents a sample, and each row represents a differentially expressed circRNA. The z-score is used to normalize the expression level of genes. Red represents higher expression, and blue represents lower expression. **(B)** C1 circRNA expression pattern, including novel_circ_002628, novel_circ_000054, and novel_circ_010008. **(C)** C2 circRNA expression pattern, including novel_circ_006817 and novel_circ_012139. **(D)** C3 circRNA expression pattern, including novel_circ_004765, novel_circ_004398, and novel_circ_011690.

With an expression fold change >2 and FDR <0.05 as the cutoff value, eight DECs were identified. The results of cluster analysis showed that the expression patterns of DECs could be divided into three categories (Figure 1A): the first category (C1) had three DECs (novel_circ_002628, novel_circ_000054, and novel_circ_010008), the second had two DECs (novel_circ_006817 and novel_circ_012139), and the third had three DECs (novel_circ_004765, novel_circ_004398, and novel_circ_011690). C1 DEC expression decreased immediately after fluvalinate administration and then gradually increased with the passage of time (Figure 1B). C2 DEC expression showed the highest expression value at 20 days after fluvalinate treatment (Figure 1C). C3 DEC expression increased immediately after fluvalinate administration and then gradually decreased to the pre-dose level (Figure 1D).

### 3.4 Structural verification and real-time quantitative PCR verification of DEC sequence and expression patterns

**FIGURE 2:**
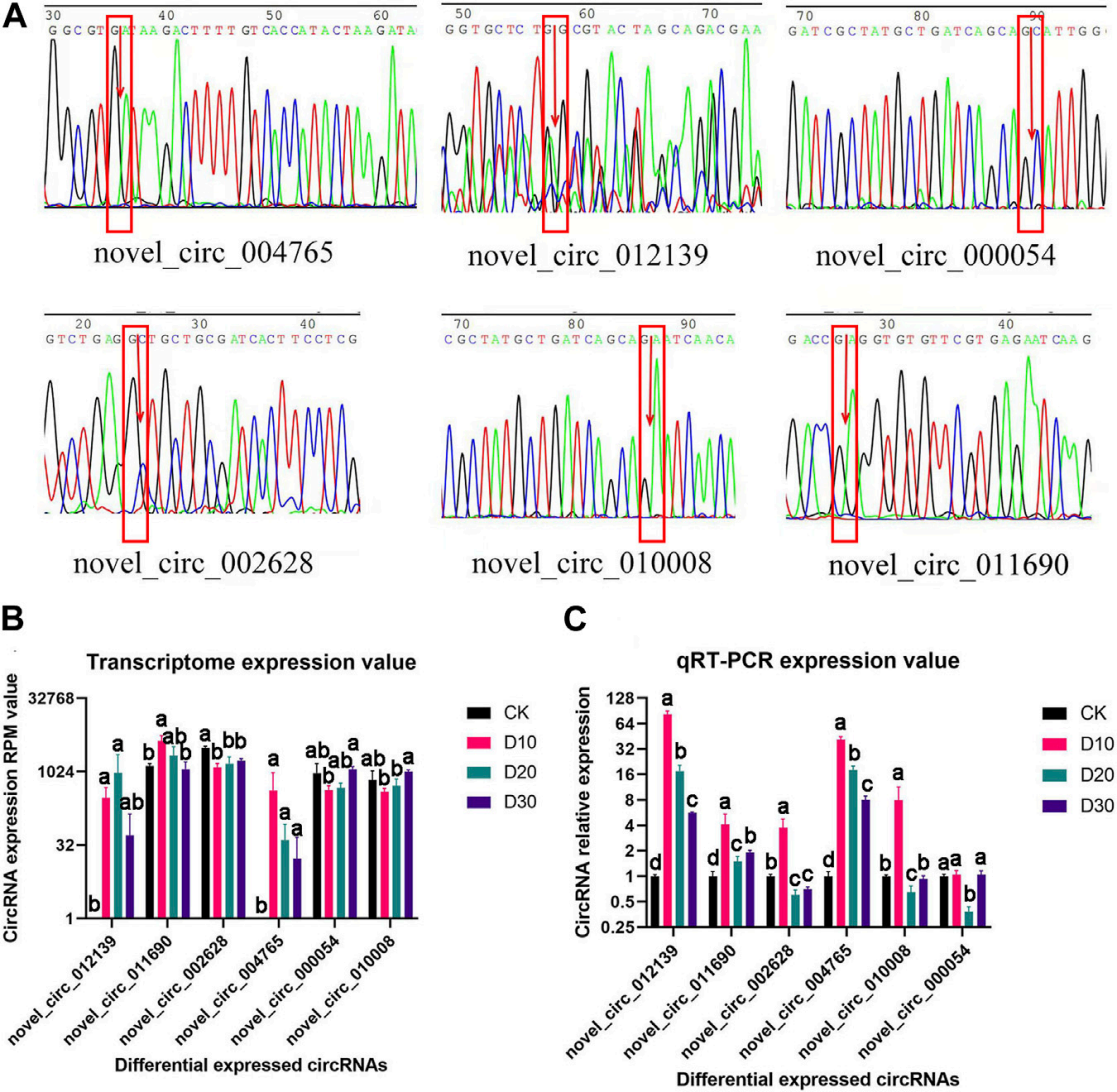
Structural verification and real-time quantitative PCR verification of DEC sequence and expression patterns. **(A)** Sequencing peak map of DECs at loop junction location; the position is marked by the red rectangle, and arrow is the loop junction. **(B)** Transcriptome expression value of six DECs. **(C)** qRT-PCR expression value of six DECs. CircRNAs received structural and significant differences which are indicated by different symbols with FDR <0.05 in RNA-Seq results and *p* < 0.05 in qRT-PCR results.

Of the eight DECs, the circular structure and sequence of six DECs were successfully verified (Figure 2A). Furthermore, qRT-PCR results proved that the expression patterns of all six DECs were basically consistent with the transcriptome sequencing results (Figures 2B, C).

### 3.5 Construction of the ceRNA network

**FIGURE 3:**
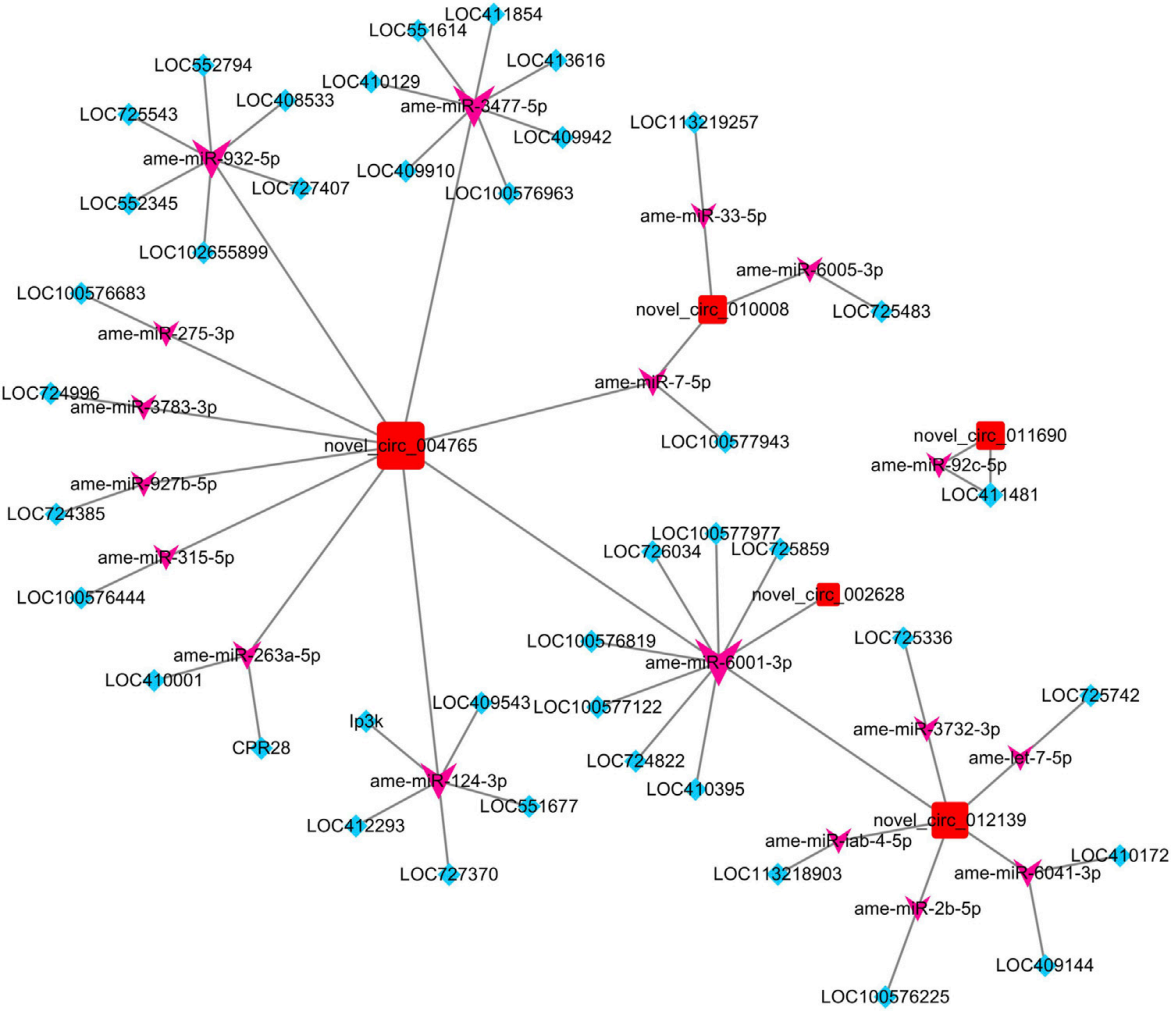
ceRNA network constructed by DECs, predicted miRNAs that can be adsorbed by DECs, and predicted targeting genes of miRNAs. The red square nodes represent circRNAs, the purple arrow nodes represent miRNAs, and the blue diamond nodes represent genes.

After taking the intersection of the results of the co-expressed gene–circRNA relationship, miRNA–circRNA targeting relationship, miRNA–mRNA targeting relationship, and miRNA–mRNA expression correlation relationship, a ceRNA network of five circRNAs, 41 genes, and 18 miRNAs was constructed (Figure 3).

To confirm the function of five DECs in the network, we performed GO enrichment on genes regulated by each DEC through the ceRNA mechanism (Table 2). The GO enrichment results showed that novel_circ_002628 may be involved in apoptosis and nucleic acid binding-related regulation, and novel_circ_012139 may have the function of regulating ion transmembrane transport. Other circRNAs failed to obtain significant enrichment results.

**TABLE 2:** Significantly enriched GO terms and KEGG pathways of genes regulated by five differentially expressed circRNAs through the ceRNA mechanism.

circRNA	GO term (TOP5)	KEGG pathway (TOP5)
novel_circ_002628	Cell death, programmed cell death, death, regulatory region DNA binding, and regulatory region nucleic acid binding	Apoptosis-multiple species and apoptosis-fly
novel_circ_012139	Regulation of transmembrane transport, regulation of ion transmembrane transport, regulation of ion transport, regulation of localization, and regulation of transport	Apoptosis-multiple species
novel_circ_010008	No significant enrichment items	No significant enrichment pathways
novel_circ_004765	No significant enrichment items	Hedgehog signaling pathway-fly, apoptosis - fly, and Wnt signaling pathway
novel_circ_011690	No significant enrichment items	mTOR signaling pathway

To further identify the biological pathways and processes that circRNAs are involved in regulating through the ceRNA mechanism, we also performed KEGG pathway analysis. Of the five DECs, four received significantly enriched pathways, except for novel_circ_010008 (Table 2). Among them, both novel_circ_002628 and novel_circ_012139 regulate LOC100576819 by adsorbing ame-miR-6001-3p, while the gene LOC100576819 was significantly enriched in the apoptosis pathway. novel_circ_011690 regulates LOC411481 by adsorbing ame-miR-92c-5p, while LOC411481 is significantly enriched in the mTOR signaling pathway. novel_circ_004765 received the most significantly enriched pathways and genes. It regulates five protein-coding genes by adsorbing ame-miR-3477-5p, ame-miR-932-5p, ame-miR-6001-3p, and ame-miR-3477-5p.

## 4 Discussion

To construct the ceRNA network, miRNA–circRNA and miRNA–mRNA targeting sites were predicted in this study. From the circRNA–miRNA interactions, we found that various circRNAs interact with different miRNAs. Among them, several circRNAs can bind to multiple miRNAs with different target sites. For example, novel_circ_004765 contains 17 potential binding sites for miRNAs, which may indicate its powerful function as a miRNA sponge. To ensure the sequence accuracy of circRNAs, we performed structural verification of link sites for all differential circRNAs. However, it should be noted that among the six differential circRNAs that were validated, the sequencing electropherogram of novel_circ_012139 always presented an excessive background that may lead to erroneous results.

To explore the functions of DECs, each circRNA in the ceRNA network was subjected to functional enrichment analysis with its related genes. The GO enrichment results showed that the predicted genes regulated by differentially expressed circRNAs are mainly enriched in cell death and ion transmembrane transport-related terms. In addition, the KEGG pathway analysis results showed that apoptosis, Hedgehog signaling pathway-fly, Wnt signaling pathway, and mTOR signaling pathway received significant enrichment.

For novel_circ_002628 and novel_circ_012139, LOC100576819 was the only gene affected by their ceRNA regulation and obtained significant enrichment in the KEGG pathway analysis. In addition, LOC100576819 was also predicted to be regulated by novel_circ_004765. Encoding a homologous protein to that of *Drosophila* caspase protein (DRONC), the function of LOC100576819 in *A. mellifera ligustica* has not been reported yet. In *Drosophila*, DRONC has been confirmed as a major initiator caspase for the programmed cell death of peptidergic neurons (Memczak et al., 2013; Qi et al., 2020) and demonstrates important functions in optic lobe development. Our previous research has proved that the expression level of LOC100576819 in the brain tissue of *A. mellifera ligustica* increased after the administration of fluvalinate and gradually decreased to the pre-dose level in the following time stages (Fischer and Raabe, 2018). During the ceRNA network, ame-miR-6001-3p was the only miRNA in the ceRNA network, and ame-miR-6001-3p targets novel_circ_002628, novel_circ_012139, and novel_circ_004765 at the same time and is also the only miRNA predicted to target LOC100576819, which may therefore play an important role in this mediating process. However, the detailed functions of miR-6001-3p are still unknown, while the only report of miR-6001 on animals is seen in a study where it was discovered as a newly identified miRNA (Rinderer et al., 1999). Furthermore, although predicted to be regulated by the ceRNA machinery of multiple circRNAs simultaneously, only LOC100576819 showed a similar expression pattern to novel_circ_004765. A possible inference is that novel_circ_004765, as the main ceRNA, regulates the expression of LOC100576819 by competitively adsorbing ame-miR-6001-3p, thereby activating the apoptotic signaling pathway and positive regulation of cell death, but there may be more complex mechanisms affecting the expression of LOC100576819. Overall, the aforementioned results and speculation are subjected to further experimental verification.

In this study, novel_circ_004765 gained the most predicted ceRNA mechanism-regulated genes, targeted miRNAs, and significantly enriched KEGG pathways. Our results showed that novel_circ_004765 may affect the apoptosis pathway by regulating LOC100576819 and LOC408533. The function of gene LOC408533 in *A. mellifera ligustica* remains unknown. LOC408533 encodes the homologous protein of ASK1 in *Drosophila*, which is orthologous to several human genes, including MAP3K15 and MAP3K5 (Saeed et al., 2003). In *Drosophila*, ASK1 has already been reported as a core kinase component of the insulin/insulin-like growth factor pathway, and it also acts as a highly sensitive sensor to activate the JNK and p38 signaling pathways when cells are damaged (Santabárbara-Ruiz et al., 2019). In *Caenorhabditis* *elegans*, ASK1 plays an important role in the Sarm1/TIR-1-ASK1/NSY-1-p38 MAPK pathway, which is closely related to the process of inhibition of axonal degeneration activated by CaMKII/UNC-43 (Shannon et al., 2003). In our previous research, CaMKII has been identified as a key gene in the post-transcriptional regulation after fluvalinate administration (Fischer and Raabe, 2018). Therefore, novel_circ_004765 may regulate the expression of LOC408533 (ASK1) gene by competitive adsorption of ame-miR-932-5p, realize the regulation of JNK, p38, and MAPK signaling pathways, and play an important role in the resistance to fluvalinate-induced injury and stress.

Our results also predicted that novel_circ_004765 can regulate genes in the Hedgehog signaling pathway (LOC410129 and LOC409942) and Wnt signaling pathway (LOC410129 and LOC409910) through the ceRNA mechanism. Casein kinase 1 (CK1) and Brother of Ihog (Boi) homologs are encoded by LOC410129 and LOC409942, respectively. CK1 regulates Hh signaling at multiple levels in the Hedgehog (Hh) pathway and is essential for neural function maintenance (Shi et al., 2014; Simon et al., 2021). Boi is part of the Ptc-co-receptor complex for Hh signaling (Tangredi et al., 2012). Its mutations have effects on eye development, neural differentiation, and wing patterning (Thölken et al., 2019). Gene LOC409910 encodes the ras-like GTP-binding protein Rho1 (Rho1), which participates in the axon regeneration of *C. elegans* and the *Drosophila* blood–brain barrier formation (Turner, 1985). Interestingly, novel_circ_004765 was predicted to regulate all three genes (LOC410129, LOC409942, and LOC409910) by adsorbing ame-miR-3477-5p. miR-3477 has only been found in three species, including honeybees, jewel wasps, and red flour beetles, and its function is unknown (Van Alphen and Fernhout, 2020). The results of expression correlation analysis and targeting relationship prediction showed that ame-miR-3477-5p can target LOC410129, LOC409942, and LOC409910, which can be used as a clue to reveal the function of miR-3477-5p and its sponge RNA, novel_circ_004765.

novel_circ_011690 was only predicted to adsorb one miRNA (ame-miR-92c-5p) and regulate one gene (LOC411481) through the ceRNA mechanism. The function of miR-92c is still unknown. LOC411481 encodes ribosomal protein S6 kinase alpha-2, RSK2, also known as S6kII. To the best of our knowledge, there is currently no research report on RSK2 and S6kII in honeybees. RSK2 has been shown to be important in the maintenance of normal neural function in *Drosophila* (Wang et al., 1996; Wieczorek et al., 2020). RSK2 is important in the development of the nervous system, and its absence can cause cognitive and emotional problems (Yu et al., 2012). Our results indicate that novel_circ_011690 may promote LOC411481 expression through competitive adsorption of ame-miR-92c-5p, which plays a role in maintaining the function of brain nerve tissue and resisting fluvalinate poisoning.

## 5 Conclusion

In summary, our results revealed the circRNA expression profile changes in the *A. mellifera ligustica* brains before and after fluvalinate treatment. A total of six circRNAs were identified as DECs and obtained structural and sequence verification, and five of them were predicted to be involved in post-transcriptional regulation of brain tissue through ceRNA regulatory mechanisms as miRNA sponges. Further functional analysis revealed that they may be involved in the regulation of apoptosis in the brain nerves of *A. mellifera ligustica* by indirectly affecting the genes in the apoptosis, Hedgehog signaling, Wnt signaling, and mTOR signaling pathways. This study improves our understanding of the expression and function of circRNAs in the brain tissue of *A. mellifera ligustica* and provides information on the role of the ceRNA mechanism in response to fluvalinate stress.

## Data Availability

The datasets presented in this study can be found in online repositories. The names of the repository/repositories and accession number(s) can be found in the article/Supplementary Material.
